# Effects of *Portulaca oleracea* L. (purslane) on the metabolic syndrome: A review

**DOI:** 10.22038/IJBMS.2022.63264.13967

**Published:** 2022-11

**Authors:** Zeinab Ebrahimian, Bibi Marjan Razavi, Seyed Ali Mousavi Shaegh, Hossein Hosseinzadeh

**Affiliations:** 1 Department of Pharmacodynamics and Toxicology, School of Pharmacy, Mashhad University of Medical Sciences, Mashhad, Iran; 2 Targeted Drug Delivery Research Center, Department of Pharmacodynamics and Toxicology, School of Pharmacy, Mashhad University of Medical Sciences, Mashhad, Iran; 3 Clinical Research Unit, Ghaem Hospital, Mashhad University of Medical Sciences, Mashhad, Iran; 4 Orthopedic Research Center, Mashhad University of Medical Sciences, Mashhad, Iran; 5 Laboratory for Microfluidics and Medical Microsystems, Bu Ali Research Institute, Mashhad University of Medical Sciences, Mashhad, Iran; 6 Pharmaceutical Research Center, Pharmaceutical Technology Institute, Mashhad University of Medical Sciences, Mashhad, Iran

**Keywords:** Diabetes mellitus, Hypertension, Metabolic syndrome, Obesity, Portulaca oleracea, Purslane

## Abstract

Metabolic syndrome (MetS) is defined as a disorder with multiple abnormalities, including obesity, high blood pressure, dyslipidemia, and high blood glucose. MetS is the best-known risk factor for type 2 diabetes mellitus (T2DM), cardiovascular disease (CVD), and obesity. With the globally increasing prevalence of MetS and its related abnormalities, attention to safe and effective prevention and treatment of this complex disorder has been increased. In particular, most treatments have been devoted to using natural agents that could provide more reliable and effective medicinal products with fewer side effects. *Portulaca oleracea* L. (purslane) is an herb whose therapeutic properties could be found in some ancient medical books. Purslane has shown analgesic, antispasmodic, skeletal muscle relaxant, bronchodilator, antiasthmatic, anti-inflammatory, antiseptic, diuretic, antibacterial, antipyretic, and wound-healing properties. In addition, purslane’s hypoglycemic and hypolipidemic properties have been reported in different studies. The positive effects of this plant include reducing stress oxidative and inflammation along with the atherogenic index, improving insulin level and glucose uptake, decreasing lipid profiles, and ameliorating weight gain. These activities could reduce MetS complications. This review aims to provide a comprehensive overview of various *in vitro*, animal, and human studies regarding the effect of *Portulaca oleracea* on metabolic syndrome to better understand the underlying mechanisms of action for designing more effective treatments.

## Introduction

Metabolic syndrome (MetS) is the clustering of multiple abnormalities so that a preclinical condition could develop into T2DM or CVD (1-3). Different people and organizations have proposed various definitions and diagnostic criteria over the past decades aimed at being easy to use in clinical settings, and all share similar diagnostic thresholds (2). The critical manifestations of MetS are dyslipidemia, raised arterial blood pressure, dysglycemia, and abdominal obesity; that obesity is more important than the other ones (1-4). Based on the National Cholesterol Education Program (NCEP) guidelines, people with three of these abnormalities are in danger of metabolic syndrome (5). People with MetS have a 46% higher mortality risk than others. Several factors, including age, physical inactivity, nutrition, lifestyle, medications, and socioeconomic conditions, have been identified as potential risk components for MetS (6). 

With the globally increasing prevalence of MetS and its related abnormalities such as obesity and hypertension, attention to safe and effective prevention and treatment of this complex disorder has been increased (7). 

Treatment of MetS could be achieved through various approaches, such as improving lifestyle (healthy diet and physical activity level), bariatric surgery, and using pharmaceutical agents (8-10). In this way, administration of herbal medicines could provide a more reliable and effective treatment strategy with fewer side effects (11). 

Many studies have been devoted to showing the effectiveness of various herbs and their active metabolites in treating MetS, including *Rosmarinus officinalis* (12), *Nigella sativa *(13), *Cinnamomum verum *(14), *Vitis vinifera* (15), *Allium sativum *(16, 17), *Persea Americana *(18), *Crocus sativus *(11, 19), *Berberis vulgaris *(20), *Capsicum annuum *L. (21), *Garcinia mangostana* (22, 23), and rutin (24).

*Portulaca oleracea* L. (purslane) belongs to the *Portulacaceae* family, which grows throughout the world (25). Purslane leaves contain many chemical compounds, including flavonoids, alkaloids, polysaccharides, and other compounds, such as essential fatty acids, sterols, minerals, and vitamins (25, 26). Studies showed that four homoisoflavonoids, including portulacanones A-D, have been isolated from purslane with cytotoxic activity on cancer *in vitro* models (25). Dopa, dopamine, and noradrenaline are other important alkaloid compounds found in purslane (27). New alkaloids of oleracein A-E were isolated from purslane. The findings of some studies showes that these compounds have antioxidant activity (25, 27). Oleracone is another alkaloid isolated from this plant that showed anti-inflammatory activity (25). In addition, purslane contains a high concentration of omega 3, which plays an important role in improving immune function, cardiac diseases, cancer, and inflammatory complications (25, 27). 

Therapeutic properties of purslane have been mentioned in some Iranian ancient medical books like the Canon Medicine by Avicenna. This plant has been recorded in Indian and Chinese medicine’s pharmacopeia and is completely known in the Traditional Medicine of Europe (25).

Some of the therapeutic benefits attributed to purslane are antispasmodic (28), diuretic (29), antimicrobial (30), wound‐healing (31), analgesic (32), gastroprotective (33), skeletal muscle relaxant (34), and bronchodilator (35). Moreover, the antihyperglycemic and antihyperlipidemic properties of purslane have been reported in animal experiments (26).

The experimental data indicate that purslane causes antihyperglycemic effects and improves glucose homeostasis without any histopathological damage. Overall, purslane appears to be safe and well-tolerated. Its therapeutic effects are associated with fewer and less severe adverse events (36-39). The critical point is that if a patient uses any medicinal plant or has a tendency to use it, he must consult with his health care providers to prevent drug interactions and adjust the dose of antidiabetic or other drugs (40).  

**Figure 1:**
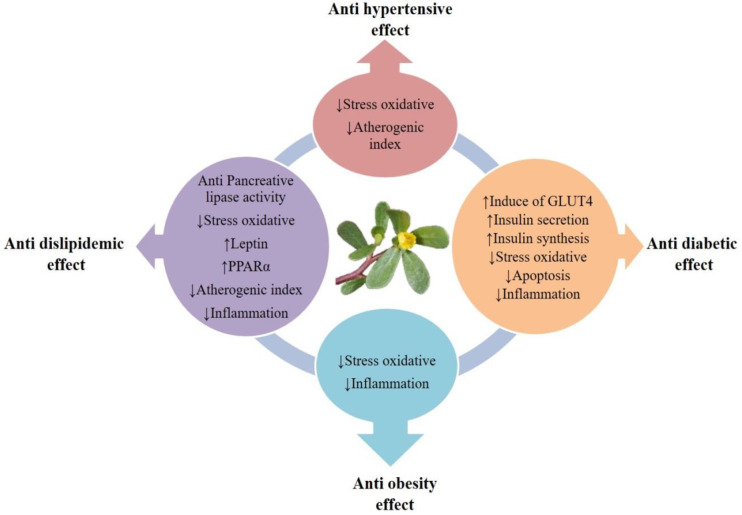
Schematic description for mechanisms of purslane (*Portulaca oleracea* L.) in ameliorating metabolic syndrome complications. Intercellular adhesion molecule (ICAM)-1, Vascular cell adhesion protein 1 (VCAM-1), Glucose transporter type 4 (GLUT4), Peroxisome proliferator-activated receptor (PPARα), Body mass index (BMI)

This article aims to review the properties and effects of purslane and its active ingredients in improving the abnormalities of metabolic syndrome, including obesity, hypertension, hyperlipidemia, and hyperglycemia, according to *in vitro*, animal, and clinical studies for better understanding and analysis of underlying mechanisms of action (Figure 1).

***Search strategy***

The literature search was conducted using Google Scholar, Pubmed, Scopus, and Web of Science electronic databases or search engines. The search terms included “*Portulaca oleracea”*, purslane, hypertension, “blood pressure,” hypotensive, antihypertensive, diabetes, hyperglycemia, insulin, hypoglycemic, antihyperglycemic, antidiabetic, “blood glucose,” dyslipidemia, hyperlipidemia, “high cholesterol,” “high triglyceride,” hypercholesterolemia, hypertriglyceridemia, atherogenic, atherosclerosis, obesity, overweight, appetite, anti-obesity, “weight loss” and “metabolic syndrome.” Only articles written in the English language and published in peer-reviewed scientific journals were considered, and duplicated articles were excluded. All articles were included if they evaluated the effect of purslane on metabolic disorders. Studies were identified through online databases from their inception up to January 2022.

***Effects on blood glucose***

Many studies show that type 2 diabetes mellitus (T2DM) is often the primary manifestation of metabolic syndrome, indicated by hyperglycemia due to insulin resistance (1, 2, 41). 

In diabetic patients, chronic hyperglycemia is associated with atherosclerosis, hypertension, microvascular complications affecting the eyes, kidneys, and nerves, and an increased risk for CVDs, leading to increasing morbidity and mortality (1, 21). Nowadays, diabetes mellitus causes death and disability in advanced and developing countries (42). According to World Health Organization (WHO) reporting in March 2013, 347 million people had diabetes globally, which will be the 7^th^ cause of death in 2030 (43, 44). Also, based on the International Diabetes Federation (IDF) estimation, 451 million adults lived with diabetes worldwide in 2017, projected to increase to 693 million by 2,045 if no preventive strategies are adopted (42). Since many studies show that some medicinal plants, such as *Abelmoschus esculentus* (45), *Ginkgo biloba* (46), and Boswellia species help the control of blood glucose levels (47) usage of herbal medicine is recommended to treat or manage diabetes. Purslane (*P. oleracea*), which reportedly has anti-diabetic properties, is a promising plant medicine used to treat diabetes mellitus for many years as folk medicine (48). The hypoglycemic properties of purslane and its constituents have been demonstrated in various studies. In this section, we summarized some studies designed to investigate the effects of purslane on plasma blood glucose.

***In vitro studies***

An investigation showed that an extract solution of purslane (*P. oleracea*) and tindora (*Cocciniagrandis*) could induce glucose transporter type 4 (GLUT4) translocation and increase intracellular glucose concentrations in insulin-sensitive CHO-K1 cells and adipocytes in an insulin-like manner (48). 

Another research was designed to study how *P. oleracea* alcoholic extract stimulates insulin release from the INS-1 pancreatic β-cells. The results showed that purslane extract at 10 to 200 μg/ml significantly enhanced insulin secretion in a dose-dependent manner. These findings also indicated that purslane extract stimulates insulin secretion through a K^+^ ATP channel-dependent pathway in INS-1 pancreatic β-cells (39). Also, an investigation indicated that 0.5 mg/ml of polysaccharides isolated from *P. oleracea* partially caused a dose-dependent inhibition of voltage-gated INa and developed insulin synthesis and cell survival in the rat's INS-1 cell line (49).

A study researched the hypoglycemic properties of purslane via inhibition of carbohydrate-hydrolyzing enzymes, α-amylase, and α-glucosidase. This study used an alcoholic extract from fresh or dried leaves of purslane. The results showed that fresh hydroalcoholic extract had the highest radical scavenging in ABTS and DPPH tests. In contrast, the dried hydroalcoholic extract displayed the highest α-glucosidase inhibitory effects (50). 

The results of another study on 3T3-L1 adipocytes suggested that the impact of extract of *P. oleracea* on glucose uptake might be via stimulating GLUT4 translocation to the membrane through activating the PI3K and AMPK pathways (51).

Another study examined glucose uptake in 3T3-L1 adipocytes by (E)-5-hydroxy-7-methoxy-3-(2′-hydroxybenzyl)-4-chromanone (HM-chromanone) which is an ingredient separated from the extract of purslane*.* The results suggested that this component may improve glucose uptake by stimulating GLUT4 translocation to the plasma membrane via activating the PI3K/AKT and AMPK pathways (52).

A recent study was carried out on the effects of HM-chromanone isolated from purslane and its possible underlying mechanisms in L6 skeletal muscle cells. The results showed that HM-chromanone promoted glucose uptake by activating the PI3K/AKT and CaMKKβ-AMPK pathways. Also, HM-chromanone increases glycogen synthesis via the GSK3 α/β pathway in these cell lines (53).

In another study, the protective effects of HM-chromanone divided from the aerial part of *P. oleracea* against glucotoxicity-induced apoptosis were investigated in INS-1 pancreatic cells that were pretreated with high glucose. This study revealed that treatment by 10-20 μM of HM-chromanone increased cell viability dose-dependently and significantly decreased the reactive oxygen species (ROS), TBARS, and nitric oxide levels (54).

The insulin secretion mechanisms induced by polysaccharides from *P. oleracea* in the INS-1 cell line have been investigated in another study. The findings indicated that polysaccharides of purslane induced insulin secretion in these cells via VGSC by alternating its action and subunit expression. They also caused alternation by following VGSC-dependent phenomena such as the change of cell membrane, mitochondrial membrane potential, intracellular calcium release, ATP metabolism, and cell survival (55).

***Animal studies***

In some *in vivo* studies, the effects of purslane and its constituents on blood glucose levels and its related factors in diabetic models have been investigated. In total, these studies show that purslane can decrease HbA1C and glucose levels and enhance insulin compared with diabetic animals. 

A study investigated the effects of seed powder of purslane on the streptozotocin-induced diabetic mouse model. In this trial, streptozotocin (STZ, 60 mg/kg) was injected intraperitoneally for 5 days to induce diabetes in mice. A week later, diabetic mice were divided into five groups receiving metformin (130 mg/kg/day) or three doses of purslane seed powder (812.5, 1,625, and 3,250 mg/kg/day) by intragastric administration for 4 weeks. This study showed that purslane could decrease fasting blood glucose and glycated hemoglobin levels in diabetic mice (56).

Also, in a study about the effect of the aqueous extract of aerial parts of *P. oleracea* (300 mg/kg/day, p.o.) on diabetic nephropathy for ten weeks in type 2 diabetic db/db mice, it was shown that purslane markedly diminished plasma creatinine and blood glucose levels compared with db/db mice (57). Also, a study that induced both hypercholesterolemia and diabetes in the same animals showed that extract of purslane leaves (1 gr per 100 gr diet, 28 days) could decrease blood glucose and HbA1C, and increase insulin levels (58). However, the findings of another study revealed that the aqueous extract of *P. oleracea *did not show any hypoglycemic activity in normal fasted and STZ-induced diabetic rats (59). 

***Clinical studies***

 In a randomized, double-blind controlled clinical trial performed on 30 type-2 diabetic cases, daily consumption of 10 g of purslane seed powder combined with regular care and exercise could significantly reduce fasting and post-prandial serum levels of blood glucose and insulin (60).

 In this regard, a double-blind study evaluated the effect of purslane with or without aerobic exercise on type 2 diabetic women for 16 weeks. The purslane groups were treated daily with capsules containing purslane seed powder (2.5 g with lunch and 5 g with dinner). Measuring blood glucose and lipid profiles showed that these items significantly diminished in purslane groups, while HDL increased significantly. Furthermore, the protein and mRNA levels of NF-κB, TIMP-1, MMP2 and MMP9, CRP, CST3, and CTSS in the patients that received purslane decreased significantly. Simultaneously, the levels of Glucagon-like peptide–1 (GLP1) and glucagon-like peptide-1 receptor (GLP1-R) increased drastically (61).

 Another double-blind study identified that receiving capsules of purslane seeds (7.5 g twice daily with food) for eight weeks could diminish glucagon-like peptide-1 (GLP-1) concentrations in type 2 diabetic women. Still, there were no changes in the concentration of GLP1-R and no relationship existed between changes in GLP1 and its receptor (62).

 A randomized placebo-controlled clinical trial on type 2 diabetic adults treated with an oral hypoglycemic agent at the beginning of the test showed that HbA1C declined in patients who received purslane (3 capsules/day; 180 mg/day) for 12 weeks (39).

 Also, a randomized controlled clinical study was done on purslane seed consumption (10 g/day) with a low-calorie diet in 54 patients with NAFLD for 8 weeks. The results revealed a significant reduction in levels of serum FBS, QUICKI, TC, and LDL-C compared with the control group (63). However, another randomized double-blinded clinical trial showed that capsules containing hydroalcoholic extract of aerial parts of purslane (300 mg/day) made no significant changes in insulin resistance and FBS in 74 patients with NAFLD for 12 weeks (64). 

 In this regard, a randomized cross-over clinical study on purslane seed consumption (10 g/day) with 240 ml low-fat yogurt revealed a slight decrease in FBS but no notable impact was observed on serum insulin levels or HOMA-IR score in 48 patients with type 2 diabetes for five weeks (65).

**Figure 2:**
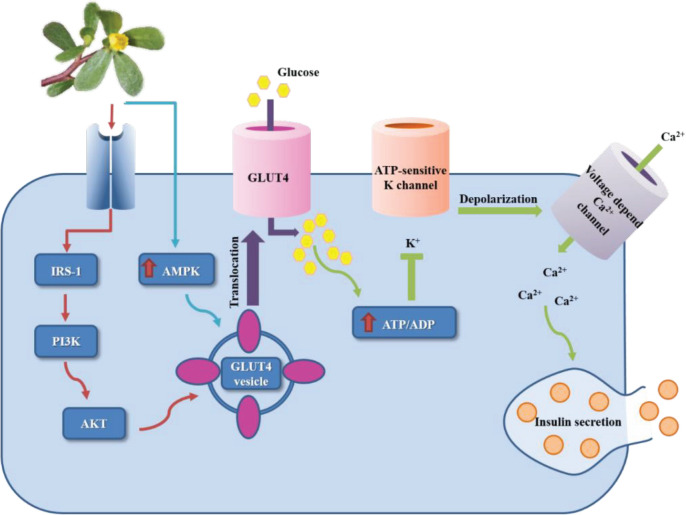
Schematic description showing the mechanisms of purslane (*Portulaca oleracea* L.) and antidiabetic effects. Insulin receptor substrate 1 (IRS-1), Phosphoinositide 3-kinases (PI3K), Protein kinase B (PKB, or AKT), AMP-activated protein kinase (AMPK), Glucose transporter type 4 (GLUT4)

In summary, *P. oleracea* L. may act as a protective or therapeutic agent for diabetes mellitus by various mechanisms, including a decrease in glucose level and HbA1C, an increase in insulin level, a decrease in insulin resistance, antioxidant and anti-inflammation activities in the animal models, and stimulation of GLUT4 translocation through activating the PI3K and AMPK pathways (Figure 2). 

***Effects on lipid profile***

Hyperlipidemia is a class of metabolic disorders described by elevated levels of TC, TG, LDL-C, and low HDL in the blood. Chronic hyperlipidemia could lead to metabolic syndrome and cardiovascular and cerebrovascular diseases (66). Since oral antihyperlipidemic drugs have some adverse effects, the use of herbal medicines has attracted more attention (67). In this section, we focused on studies that investigated the effects of purslane on lipid profiles.

***In vitro studies***

The anti-lipase property of extracts of aerial parts of* P. oleracea*,* Urtica urens*, *Lathyrus hierosolymitanus, and Brassica, *considered traditional Palestinian herbs, was investigated on porcine pancreatic lipase type II inhibition. Pancreatic lipase enzymes are vital in the digestion and metabolism of dietary lipids. The findings proved the anti-lipase activity of* U. urens, P. oleracea, and B. napus* so that these herbal extracts could be used as anti-lipase agents or food additives to minimize fat absorption in diets (68).

In another study, the inhibitory action of pancreatic lipase of 18 species of edible plants in the Calabria region (Italy) was assayed through *in vitro* tests. The results showed that the hydroalcoholic extract of purslane leaves had the most potent pancreatic lipase type II inhibitory effect among these plants (69).

A recent study was carried out on the effect of HM-chromanone, isolated from *P. oleracea*, on 3T3-L1 adipocytes. Findings showed that HM-chromanone decreases adipocyte differentiation, TG accumulation, and leptin, and increases glycerol and adiponectin secretion in this cell line. Also, this research suggests that HM-chromanone acts as an anti-adipogenesis agent by suppressing adipogenic transcription factors and activating of AMPK (70).

***Animal studies***

Many studies have investigated the antihyperlipidemic and antidiabetic activities of *P. oleracea* on various animal models of hyperlipidemia and diabetes and have provided scientific evidence for clinical use. These investigations have revealed that purslane could modify and decrease lipid profiles in hyperlipidemic animals. The details of these studies are presented in Table 1.

The hypolipidemic effects of purslane seed extracts have been evaluated in STZ-induced diabetic rats. The results revealed a significant effect in decreasing serum TGs’ higher levels compared with the STZ group. The findings of this study powerfully exhibit the potential of the non-polar extract of purslane seed against hyperlipidemia in diabetic conditions. The rapidity of onset, more prolonged duration, and most efficacy for the atherogenic index belong to the ethyl acetate extract. Also, the extract of methylene chloride (DCM) had the maximum impact on diminishing the LDL-C level (96).

***Clinical trial***

A study investigated the effects of purslane seeds on biomarkers of oxidative stress in 40 T2DM patients by a cross-over randomized controlled clinical trial. These people received either 10 g/day seeds of purslane with 240 ml low-fat yogurt or only 240 ml low-fat yogurt for 5 weeks. After two weeks of washout, patients were transferred to the other group for an additional 5 weeks. Measurement of biomarkers of oxidative stress in fasting blood samples at the baseline and final study period did not show any change in malondialdehyde, antioxidant potential, or oxLDL (65). A triple-blinded randomized controlled trial studied the anti-dyslipidemic effects of *P. oleracea* on obese adolescents. The case group received capsules of purslane seeds (500 mg, Bid) for one month, while the control group was given placebo (lactose) capsules in the same way. The results showed that TG, LDL-C, and TC significantly decreased in the purslane group (97).

Another randomized, double-blind controlled clinical study was carried out to examine the anti-diabetic property of purslane seeds on thirty T2DM subjects. A group of patients took 5 gr of purslane seeds (Bid) and another group took metformin (1500 mg/day). The findings revealed a significant decrease in serum fasting and postprandial blood glucose, insulin, TC, LDL-C, TG levels, BMI, and body weight. Moreover, the results showed a significant increase in HDL-C and albumin and a nonsignificant change in ALP level in the purslane-treated group. Except for LDL-C, HDL-C, and ALP, similar results were obtained for the metformin group (60).

 Consequently, consuming daily capsules of purslane seed powder (2.5 g with lunch and 5 g with dinner) and aerobic exercise were investigated in a double-blind study on 196 T2DM women at 16 weeks. The results revealed that the blood glucose and lipid profiles significantly declined in the purslane groups, while HDL enhanced considerably. This study suggested that purslane seeds, without or with physical activity, could modify atherosclerosis plaque biomarkers in T2DM (61). The findings of another randomized, placebo-controlled clinical research revealed that daily usage of three capsules of purslane extract (180 mg/day) for 12 weeks caused a significant decrease in serum TC in T2DM patients that received a single dose of a hypoglycemic agent at the beginning of the trial (39).

 A randomized, double-blind controlled study investigated the impacts of Persian medicinal (PM) capsules, including dried fenugreek, sumac, and purslane, on lipid profile in 74 dyslipidemic patients for 6 weeks. This clinical trial showed that PM significantly reduced serum cholesterol and LDL levels compared with the placebo group. Moreover, TG and VLDL levels decreased using the PM caps but could not significantly increase HDL levels (98).

Another randomized controlled clinical trial on 54 NAFLD patients was carried out to study the effects of purslane seeds (10 g/day) with a low-calorie diet for 8 weeks. The results revealed a significant decrease in TC and LDL-C levels in serum (63). However, a placebo-controlled, double-blind, randomized clinical trial displayed the capsules filled with hydroalcoholic extract of aerial parts of purslane (300 mg) showed no significant changes in TG and LDL-C in 74 patients with NAFLD for 12 weeks (64). 

In another trial, lovastatin therapy and the effects of purslane on serum lipids, lipoproteins, and PON1 activity were compared in 93 patients with an LDL-C >120 mg/dl for 45 days. The results showed that receiving purslane (50 gr/day of fresh leaves and stems) or lovastatin (20 mg/day) decreased the serum levels of LDL-C, cholesterol, and oxLDL and increased PON1 activity, ApoA1, and HDL. TG level and BMI were diminished only in the purslane group, and ApoB decreased only after taking lovastatin. This clinical study suggested purslane could reduce cardiovascular risk factors and increase PON1 activity more than lovastatin (99).

Based on the studies mentioned above, it could be inferred that purslane has hypolipidemic effects in animal models and clinical trials owing to increasing HDL and decreasing levels of TC, LDL-C, and TG. Different mechanisms including increasing antioxidant enzyme activity, decreasing lipid peroxidation, inhibiting lipid metabolism, increasing liver leptin and PPARα, and decreasing liver FAS mRNA levels are involved in the antihyperlipidemic effects of purslane (71).

***Effects on obesity***

One of the most widespread metabolic abnormalities is obesity (100). The McKinsey Global Institute announced that approximately 30% of the global population is overweight or obese, and it will reach 41% by 2030 (101). WHO defined obesity as an excessive fat mass with a BMI of ≥ 30 kg/m^2^ causes health problems. Obesity is the leading risk factor for metabolic diseases such as T2DM, hypertension, and fatty liver disorder (102). There are several methods for the management of obesity such as diet, exercise, and medication (17). Since anti-obesity drugs cause unfavorable effects, scientists have considered beneficial herbal compounds to treat and manage obesity and found some potential plants and their components that exhibit suitable anti-obesity effects (18, 20, 103-105). Studies are looking at purslane treatment to improve weight gain, but results have been conflicting depending on the models studied and outcome measures used.

***Animal studies***

 A study indicated that the six-week feeding mixture of pumpkin and purslane seeds (used as ω-6 fatty acids and ω-3 fatty acids rich sources in a ratio of 5/1) along a cholesterol-enriched diet significantly reduced body weight and relative organ weight in hypercholesterolemic rats (77). Another study investigated three types of preparations of *P. oleracea* stems as supplements with HFD diet (powder 10% with HFD diet, aqueous, and ethanolic extract 1 g/weight/day orally, 8 weeks) in hyperlipidemic rats. The results showed a considerable reduction in daily weight gain and food consumption compared with the hyperlipidemic rats fed only high-fat diets (38). 

 On the contrary, a study on dexamethasone-induced hypertension in rats showed that oral extract of P.oleracea seeds for 4 days before initiation of 14 days of dexamethasone administration did not affect rats’ weight gain (106). Another study has shown that body weight and food consumption had no differences in hypercholesterolemic rats who received only a cholesterol-enriched diet or with an aqueous extract of leaves of *P. oleracea* (0.5%) for 4 weeks (75). Another study showed that the oral treatment of alcoholic extracts of stems and leaves of purslane (200 mg/kg for 3 weeks) did not alter the body or ovarian and uterine weight in D-galactose-induced aging NMRI female mice (36). In this context, the research identified that orally receiving 100, 200, and 400 mg/kg polysaccharides extracted from aerial parts of purslane for 30 days could not affect body weight in acute exercise-induced oxidative stress in male rats (107).

On the other hand, several studies showed that purslane and its components could modify or elevate body weight in diabetic animals; nevertheless, purslane wasn’t able to reach the diabetic group’s weight to that of the control group. Alloxan or STZ induced a significant reduction in body mass in these studies (79, 84, 86-88, 91). For example, a study in alloxan-induced diabetic rats showed that oral pretreatment by extract of aerial parts of purslane extract (250 mg/kg for four weeks) could normalize body weight. However, in nondiabetic rats, it caused a significant decrease in body weight (85). Also, some studies showed that purslane extract could attenuate weight loss in a mouse model of DSS-induced colitis and LPS-induced inflammatory responses (108-110). In hypercholesterolemic animal models, purslane showed different effects on weight gain. A study on STZ-induced diabetic rats with a diet enriched with cholesterol revealed that the aqueous extract of *P. oleracea* leaves (1% in diet, 28 days) induced a significant addition in final body weight compared with control (58). 

***Clinical studies***

In a randomized, double-blind controlled clinical trial, receiving *P. oleracea* seeds powder (10 g/40 ml of skimmed yogurt; Bid) for 8 weeks significantly decreased BMI and weight in type 2 diabetic obese patients to the pre-obesity category of the WHO classification (60). Another randomized, double-blind, placebo-controlled clinical study revealed that purslane extract (three capsules: 180 mg/day) for 12 weeks decreased BMI, body weight, and HOMA-IR in adult T2DM patients receiving an oral hypoglycemic agent at the beginning (39). Also, a randomized cross-over study showed a reduction in BMI and weight following purslane seeds consumption (10 g/day with 240 ml low-fat yogurt) in type 2 diabetic cases for 5 weeks (65). Furthermore, another clinical trial showed that a 45-days diet with 50 gr/day of fresh leaves and purslane stems decreased serum levels of LDL-C, Ox-LDL, cholesterol, TG, and BMI in subjects with LDL-C > 120 mg/dl (99). 

Overall, these mentioned studies suggest that purslane could decrease body weight in clinical trials and modulate body weights in animal studies. Further studies are needed because of some contradictory results, especially in animal models and limited clinical trials.

***Effects on hypertension***

Hypertension, one of the main markers of metabolic syndrome, is a prevalent disorder associated with life-threatening complications such as kidney damage and CVDs, such as peripheral vascular and coronary artery disorders, heart failure, and stroke (111). In this session, we summarized a few studies that were performed to assess the impacts of purslane and its ingredients on blood pressure.

***Animal studies***

In a research project, the aqueous extract of aerial parts of *P*.* oleracea *(300 mg/kg/day, p.o.) in the db/db mice for ten weeks showed a marked decrease in SBP levels compared with untreated db/db mice during the experimental period. The impairment of Ach and SNP-induced vascular relaxation of aortic rings was ameliorated by diabetic db/db mice that received the purslane treatment. While compared with wild-type mice, untreated db/db mice showed significantly increased expressions of ICAM-1, VCAM-1, and E-selectin, db/db mice treated with purslane showed a reduction in these parameters (57).

According to a study on spontaneously hypertensive rats, six weeks of a diet containing purslane with a 25:1 ω-6: ω-3 fatty acid ratio could decrease SBP (74).

 Contrary to two previous studies, a survey on aqueous extract of *P. oleracea* leaves and stems (1.4-56 mg/kg, IV) showed a dose-dependent increase in rat mean arterial blood pressure (MAP) and contraction in the isolated rabbit aorta. These findings suggested that the extract by interaction with transmembrane calcium influx might affect postsynaptic α receptors. It seems the extract’s contraction response may be due to an inotropic action on the heart mediated by β receptors (112). 

 Another research identified the hydroalcoholic extract of *P. oleracea *seeds (100, 200, and 400 mg/kg/day, p.o.) for 18 days in the dexamethasone-induced hypertensive rats not only could not decrease SBP but also increased heart rate by the high dose. The authors recommended some compounds of purslane, such as noradrenaline, are the reason for these effects (106).

***Clinical studies***

 A randomized controlled cross-over clinical trial on type 2 diabetes mellitus (T2DM) patients showed oral consumption of purslane seeds (10 g/day with 240 ml low-fat yogurt) for 5 weeks decreased systolic and diastolic blood pressure (65).

Besides, a randomized, double-blind placebo-controlled clinical trial showed purslane capsules (3×60 mg/day; 180 mg/kg) significantly reduced SBP in T2DM subjects compared with the placebo group for 12 weeks (39). 

Conversely, a randomized, double-blind clinical trial in 74 patients with NAFLD displayed aerial parts of purslane hydroalcoholic extract capsules (300 mg) had no significant changes in blood pressure for 12 weeks (64). 

In summary, there are few studies to assess the effects of purslane and its components on hypertension. Among these studies, purslane could reduce SBP in clinical trials. Because of contradictory results in animal models and and limited clinical trials, further studies should be performed.

**Table 1:** Summary of the animal studies of purslane (*Portulaca oleracea* L.) and its active compounds on lipid profiles

**Study design**	**Rout of exposure /dose/constituents**	**Results**	**Mechanisms**	**References**
High fat diet-induced oxidative injury in mice	Aqueous extract of the aerial parts (100 and 200 mg/kg, orally)	Antioxidant effect Hypolipidemic effect	↓ Blood TBARS level ↓ Serum TG, LDL-c and TC levels and by ↑ HDL-C concentration ↓ Lipid peroxidation levels of blood and liver ↑ Antioxidant enzyme activities of blood and liver ↑ Leptin/b-actin (B), and PPARa/b-actin of liver ↓ Protein expression levels of liver, spleen FAS mRNA, p-PERK, and p-PERK/PERK	(71)
Dexamethasone-induced hyperlipidemia in rat	Ethanolic extract of leaves (200 and 400 mg/kg, orally)	Hypolipidemic effect Anti-Atherogenic effect	↓ TC, LDL-C, and TG ↓ Atherogenic index	(72)
Rabbits fed with a hypercholesterolemic (0.5%) diet	Hydroalcoholic extract of leaves (200, 400, and 600 mg/Kg, orally) 12 weeks	Hypolipidemic effect	↓ Serum TC and LDL-C	(73)
Spontaneously hypertensive rats (SHRs) fed diets with a 25:1 ω-6: ω-3 fatty acid ratio (FAR)	Diets containing green leafy vegetables (GLVs) including 4% (dried weight) collard greens (CG), purslane (PL), orange flesh, and sweet potato greens (SPG) 6 weeks	Hypolipidemic effect	↓ SBP ↓ Plasma adiponectin, total, and LDL-C ↑ hsCRP ↓ TG levels following the consumption of the CG (collard greens) diet ↓ not significant, TC, LDL-C, and VLDL-C levels ↑ HDL-C was increased among SHRs consuming the CG and PL diets	(74)
Hypercholesterolemic rats (1% cholesterol-enriched diet)	Aqueous extract of leaves (0.5% diet supplemented) for four weeks	Hypolipidemic effect anti-atherogenic effects	↓ TC, TG, and liver TG values ↓ Cholesterol concentrations in LDL-HDL1, HDL2, and HDL3 ↑ HDL2	(75)
Radiation-induced damage rats (single dose of 6 Gy gamma rays)	Extract stem and leaves (400 mg/kg) or fish oil (60 mg/kg) and their co-treatment (15 days)	Hepatoprotective effect Renoprotective effect Antioxidative effect	Attenuated lipids alteration, liver and kidney functions as well as oxidative stress in irradiated rats	(76)
Rats fed high cholesterol diets (cholesterol at a dose level of 2 g/100 g diet)	Flax/pumpkin or purslane/pumpkin seed mixture ratio of (5/1) (ω-3 and ω-6).	Anti-atherogenic effect Immunomodulator effect	↓ lipid parameters Improvement in IgG and IgM levels	(77)
Dietary induced hyperlipidemic rats (20% fat,1%cholesterol, and 0.25% colic acid)	Stem powder (POS-powder), stem infusion (POS-infusion) and stem 70% ethanolic extract (POS-ethanolic 70%) for 8 weeks	Hypolipidemic effect Hepatoprotective effect	Improved abnormal lipid parameters and risk ratio Ameliorated the abnormalities in the liver status of hyperlipidemic rats Improvement Liver histology	(38)
Diabetic nephropathy accelerated by renal fibrosis and inflammation in type 2 diabetic db/db mice	Aqueous extract of aerial part (300 mg/kg/day, orally) for ten weeks	Hypoglycemic effect Renoprotective effect	↓ Blood glucose and plasma creatinine level ↓ Decreased water intake and urine volume ↓ Expressions of TGF-β1, AGE, and (ICAM)-1	(78)
Fasting normal and STZ-induced diabetic rats	Aqueous extracts of *Syzygium cumini* Linn. Gymnema sylvestre (Retz.) Schult., and *P. oleracea* Linn. seeds (200 mg/kg)	------	*P. oleracea* did not show any hypoglycemic activity	(59)
STZ-induced diabetic rats	Extracted crude polysaccharide from aerial part (100, 200, and 400 mg/kg, orally)	Anti-diabetic effects Antioxidant effects Anti-inflammatory effects	↑ The body weight Improved glucose tolerance in diabetic rats ↓ FBG ↑ FINS and ISI value ↓ TNF-α and IL-6 levels ↓ MDA and SOD activities in the liver	(79)
STZ-induced rats	Extracted Polysaccharide from seeds (25 and 50 mg/kg, orally) 3 weeks	Hypoglycemic effect	↓ Blood glucose, TBARS ↑ GSH levels and GPx activity Reversed CAT and SOD near to normal values	(80)
STZ-induced diabetes rats	Aqueous mixture of extracts of *Spilanthes* *africana*; *P. oleracea*; and *Sida rhombifolia* leaves (1: 1: 1) for 21 days	Hypoglycemic effect Antioxidant effect Hypolipidemic effects	↑ HDL-cholesterol, glutathione, and TAOS ↓ Atherogenic indexes CT/HDL and LDL/HDL Scavenging property on DPPH and OH radicals	(81)
STZ-induced diabetic rats	Aqueous extract (100, 200, and 400 mg/kg/d, IP) 4 weeks	Hypoglycemic effect Antioxidant effect Anti-inflammation effect	Ameliorated glucose, MDA, IL-6, TNF-a, GSH, and TAS levels	(82)
Insulin-resistant HepG2 cells and STZ-induced C57BL/6J diabetic mice	Fresh and dried *P. oleracea* orally	Anti-diabetic effect Antioxidant effect	In insulin-resistant HepG2 cells: ↑ extracellular glucose (fresh extract > dried extract) In STZ-induced C57BL/ 6J diabetic mice: ↓ FBG Improved OGTT ↑ Insulin secretion and anti-oxidative activities Fresh extract showed stronger antidiabetic activity Relative contents of polyphenols and alkaloids (fresh herbs > the dried)	(83)
Alloxan-induced diabetic rats	Hydroalcoholic extract (whole plant) (100 and 200 mg/kg, orally)	Antidiabetic and hypolipidemic effects	↓ Blood glucose levels ↓ Serum TC, TG, and LDL-C levels ↑ Bodyweight	(84)
Alloxan-induced diabetic rats	Extract of aerial part (250 mg/kg, orally) for 4 weeks	Hypoglycemic effect	↓ HbA1C, serum levels of glucose, TNF-α, and IL-6 ↑ C peptide and insulin Improvement of the destructive effect on pancreatic islet cells	(85)
Alloxan-induced diabetic rats	Extract (200 and 400 mg/kg, orally) for 28 days	Hypoglycemic effect Hypolipidemic effect	↓ FBG, TC, and TG Improvement of body weight gain ↑ Insulin levels ↓ Blood glucose and lipid	(86)
Alloxan-induced diabetic mice	Crude polysaccharide (200 and 400 mg/kg, orally) for 28 days	Hypoglycemic effect Hypolipidemic effect	↓ FBG, TC, and TG ↑ HDL-C, body weight, and serum insulin level	(87)
STZ-induced diabetic rats	QursTabasheer 50, 100, and 200 mg/kg, orally) for 28 days	Hypoglycemic effect Hypolipidemic effect	↓ Serum glucose, TC, TG, glucose-6-phosphatase, and fructose-1-6-biphosphatase ↑ HDL-C and hexokinase	(88)
Forced swimming mice	polysaccharides of whole plant (100, 200, and 400 mg/kg, orally) for 28 days	Improved exercise endurance Antioxidant effect	↓ BLA ↑ Exhaustive swimming time ↓ MDA levels ↑ Blood glucose levels ↑ SOD, GPx, CAT levels	(89)
db/db mice	Aqueous extract of aerial parts (300 mg/kg/day, orally) for 10 weeks	Hypoglycemic effect Hypolipidemic effect Anti-inflammatory effect Hypotensive effect	↓ Blood glucose, plasma TG, LDL-C ↓ SBP ↑ Plasma level of HDL-C and insulin level Ameliorated the impairment of ACh- and SNP-induced vascular relaxation of aortic rings Suppressed overexpression of VCAM-1, ICAM-1, E-selectin, MMP-2, and ET-1 in aortic tissues Increased the insulin immunoreactivity of the pancreatic islets	(57)
Streptozotocin-induced diabetic rat	Aqueous extract of leaves (1g/kg (0.1%) with casein diet supplemented) for 4 weeks	Hypolipidemic effect Hypoglycemic effect Antioxidant effect	↓ Blood glucose, HbA1C, TC, (LDL-HDL1-C) ↑ HDL-C ↓ TG and PL ↓ TC/HDL-C and LDL-HDL1-C/HDL-C Improved PON1 and LCAT activities	(90)
Streptozotocin-induced diabetes in mice	Hydroalcoholic extract (100 and 200 mg/kg, orally) 28 days	Hypoglycemic effect Hypolipidemic effect	↑ Bodyweight and ↓ food intake ↓ Glucose, AST, ALT, TG, TC, IL-6, IL-1β, and TNFα in serum ↑ Serum insulin Alleviated pathological liver changes in diabetic mice Restored the levels of Rho–NFκB signaling-related proteins	(91)
STZ-induced diabetic rats on a 1% cholesterol-enriched diet.	Supplemented with 1% of aqueous extract of leaves, during 28 days	Hypolipidemic effect Hypoglycemic effect	↓ Glucose, HbA1C levels ↑ Insulin ↓ Plasma values of TC, TG, VLDL-C, and LDL-C ↑ HDL-C ↓ Atherogenic indices TC/HDL-C and LDL-C/HDL-C ↓ Lipid peroxidation in the liver, heart, and aorta ↑ Antioxidant enzymes activities	(58)
Alloxan-induced diabetic mice	Polysaccharides of the whole plant (200 and 400 mg/ kg) for 28 days	Hypoglycemic effect Hypolipidemic effect	↓ FBG, TC, and TG ↑ HDL-C and serum insulin level	(92)
Streptozotocin diabetes induced in ovariectomized rats	Aqueous extract (300 mg/kg, orally) for 35 days	Anxiolytic effects Hypoglycemic effect	↓ FBS Improved the spatial cognitive performance at training trials in MWM Improved the motor deficit in EPM Improved non-functional masticatory activity in TPS	(93)
Diabetic rat with fructose mixed with top feed (50% fructose + 50% top-fed)	Aqueous extract (200 and 400 mg/kg, orally) for two weeks	Hypoglycemic effect	↑ Glucose tolerance	(94)
High-fat diet-induced obese C57BL/6 mice	*P. oleracea* powder mixture with a high-fat diet (5%, 10%) for 12 weeks	Hypolipidemic effect Antiobesity	↓ Glucose, HbA1C levels ↓ Insulin ↓ Plasma values of TC, TG, TG/HDL-cholesterol index, VLDL-C, and LDL-C ↓ AI, CRF ↑ HDL-C ↓ ALT	(95)

Advanced glycation end products (AGE), Atherogenic Index (AI), Alanine aminotransferase (ALT), Anti-aspartate aminotransferase (AST), Blood lactic acid (BLA), Catalase (CAT), Cardiac risk factor (CRF), Elevated plus maze (EPM), Fasting blood glucose (FBG), Fasting serum insulin (FINS), Glutathione (GSH), Glutathione peroxidase (GPx), High-density lipoprotein cholesterol (HDL -C), Insulin sensitivity index (ISI), Intercellular adhesion molecule (ICAM)-1, Vascular cell adhesion protein 1 (VCAM-1), Lecithin: cholesterol acyltransferase (LCAT), Morris water maze (MWM), Oral glucose tolerance test (OGTT), Paraoxonase 1 (PON1), Phospholipids (PL), Superoxide dismutase (SOD), Tail pinch stressor (TPS), Thiobarbituric Acid Reactive Substances (TBARS), Total cholesterol (TC), Transforming growth factor-β1 (TGF-β1), systolic blood pressure (SBP), Triacylglycerols (TG), Very low and low-density lipoprotein cholesterol (VLDL-C, LDL-C)

## Conclusion

MetS is a tremendous and increasing global health concern known as disruptions of insulin, glucose, and lipid metabolism, obesity, and hypertension. Since this syndrome can raise the risk of T2DM and CVD, exploring novel solutions with high efficacy and minor unfavorable effects is necessary. This article reviewed and discussed findings from *in vitro* and *in vivo* studies and clinical trials to evaluate some effects of *P. oleracea *(purslane) and its active constituents on blood glucose, lipid profiles, weight gain, and blood pressure. Findings show that purslane has useful consequences on blood glucose levels through antioxidant and anti-inflammatory activities, improvement in insulin levels, a decrease in glucose and HbA1C levels, decreased insulin resistance in animal models, and stimulation of GLUT4 translocation through activating the PI3K and AMPK pathway in some *in vitro* studies. Moreover, purslane exhibits hypolipidemic effects in animal models and clinical trial studies by increasing HDL levels and decreasing TG, LDL-C, and TC levels. Anti-obesity and antihypertensive effects of this plant were reported in clinical studies; however, findings from *in vivo* studies are controversial. Thus, more studies are required to explore the effects of purslane on weight gain and blood pressure. Finally, this review concludes that *P. oleracea* and its main constituents might help with treating metabolic syndrome.

## Authors’ Contributions

HH recommended the Review topic; ZE collected the articles, processed data, and prepared the draft of the manuscript; HH and BMR supervised, directed, and managed the study; HH, BMR, and SAMS edited the article; ZE, BMR, SAMS, and HH approved the final version to be published.

## Conflicts of Interest

The authors declare no conflicts of interest.

## Ethical Review

This study does not involve any human or animal testing.
